# The most critically injured polytrauma patient mortality: should it be a measurement of trauma system performance?

**DOI:** 10.1007/s00068-022-02073-z

**Published:** 2022-08-18

**Authors:** Benjamin Maurice Hardy, Natalie Enninghorst, Kate Louise King, Zsolt Janos Balogh

**Affiliations:** 1 Department of Traumatology, John Hunter Hospital and University of Newcastle, Newcastle, NSW 2310 Australia; 2 University of Newcastle, Newcastle, NSW Australia

**Keywords:** Polytrauma, Trauma, Multiple trauma, Trauma center, Trauma care, Trauma surgery

## Abstract

**Purpose:**

The risk of death after traumatic injury in developed trauma systems is at an all-time low. Among ‘major trauma’ patients (injury severity score, ISS > 15), the risk of dying is less than 10%. This group contains critical polytrauma patients (ISS 50–75), with high risks of death. We hypothesized that the reduction in trauma mortality was driven by reduction in moderate injury severity and that death from critical polytrauma remained persistently high.

**Methods:**

A 20-year retrospective analysis ending December 2021 of a Level-1 trauma center’s registry was performed on all trauma patients admitted with ISS > 15. Patients’ demographics, injury severity and outcomes were collected. Multivariate logistic regression analysis was performed. Mortality was examined for the entire study group and separately for the subset of critical polytrauma patients (ISS 50–75).

**Results:**

A total of 8462 severely injured (ISS > 15) trauma patients were identified during the 20-year period. Of these 238 (2.8%) were critical polytrauma patients (ISS 50–75). ISS > 15 mortality decreased from 11.3 to 9.4% over the study period (Adjusted OR 0.98, 0.97–0.99). ISS 50–75 mortality did not change significantly (46.2–60.0%), adjusted OR 0.96, 0.92–1.00).

**Conclusion:**

The improvement in trauma mortality over the past 20 years has not been experienced equally. The ISS50-75 critical polytrauma mortality is a practical group to capture. It could be a group for deeper study and reporting to drive improvement.

## Background

The outcomes of major trauma patients have continued to improve since organized trauma care began [1, 2]. For practical and statistical reasons, the major trauma population has often been described as an injury severity score (ISS) of greater than 15 [3, 4]. Outcome measures in trauma care span crude mortality rate to detailed functional outcomes [2, 5, 6]. Developed trauma systems now routinely report very low mortality rates in major trauma, which definition in some countries extended to include ISS > 12 [7].

ISS, the sum of three squared numbers, is not a continuous scale and mortality does not rise monotonically throughout the spectrum of ISS but with a series of stepwise increases. This yields natural groups with similar mortality [4]. These subgroups allow for comparison between systems [8]. Although in few studies, ISS 50–75 has been demonstrated as a potential practical group of for monitoring the most critically injured patients [4, 8], a small proportion of the overall major trauma population, whose outcomes may be obscured improvements in outcomes of less severely injured patients. By definition, these groups, apart from some non-survivable single system injuries with ISS of 75, uniformly consist of poly-trauma patients with Abbreviated Injury Scale greater than two in at least two body regions [4]. We hypothesized that the reduction in mortality rate since the organization of the trauma service in a Level-1 trauma center’s population was driven by improvements in the less severely injured population, with a persistently high mortality rate among ISS 50–75 poly-trauma group.

## Methods

The John Hunter Hospital is a level 1 trauma center located in New South Wales (NSW), Australia. It is the highest volume trauma center in the state of NSW. It cared for 513 ISS > 15 major trauma patients in 2019 [9]. The trauma center is the referral center for major trauma for approximately 1.1 million people. Since 2002 all patients with ISS > 15 have been collected in a trauma registry.

**Fig. 1:**
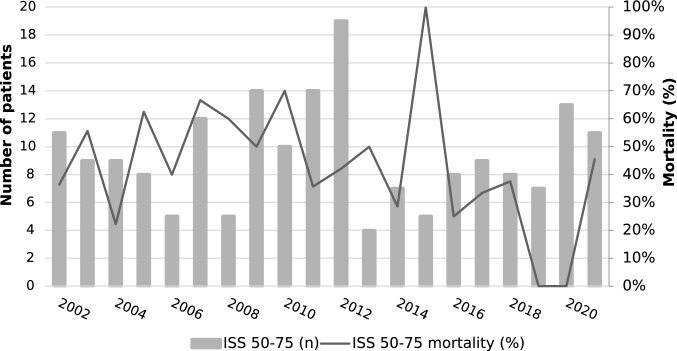
Number of and mortality of ISS > 15 patients over the study period

Data were extracted from the prospectively maintained trauma database for the entire period between January 1, 2002 and December 31, 2021. Data extracted were age, sex, blunt or penetrating mechanism, presentation type (transfer vs direct), ISS, date of injury and in-hospital mortality. Ethical approval was granted by the Hunter New England Human Research Ethics Committee with reference AU202105-03 (see Fig. 1).

Data were analyzed using Stata 17 (StataCorp. 2022. Stata Statistical Software: Release 17. College Station, TX: StataCorp LP.). Continuous data were presented as median (IQR) and categorical data as counts and proportions. Comparison between groups was with the Kruskal–Wallis test. Binary logistic regression was used to control for age, sex, injury mechanism, and interhospital transfer, and to measure interactions between variables. They were reported as odds ratios and 95% confidence intervals. Male sex was used as the reference group in the regression. Age was inserted as an indicator categorical variable, given its non-linear influence on risk of death with a threshold of 55 years of age in keeping with TRISS. Date of injury was divided into calendar year and inserted into the model as an ordinal variable over the study period. Mechanism of injury and transfer status were also included as indicator variables. Proportions were compared using Fisher’s exact test. Statistical significance was set at 5%. We used the STROBE cohort checklist when writing the manuscript [10].

**Fig. 2:**
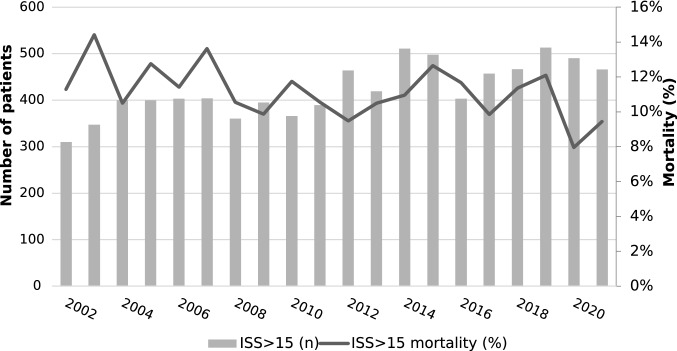
Number of and mortality of ISS 50–75 patients over the study period

## Results

There were 8,462 patients with ISS > 15 (median ISS: 20, IQR: 17–26) during the 20-year study period of whom were 72.1% male and 27.8% were female with an overall mortality of 11.1%. There were 238 patients with an ISS 50–75 (12 patients/year, 2.8% of the total ISS > 15 population, median ISS: 57, IQR: 50–66, mean ISS: 58) of whom 74.3% were male and 25.6 were female with a mortality of 51.7% (Table 1). The mean age increased over the study period (0.5 years/year, *p* < 0.001). The overall ISS > 15 mortality rate improved over the study period (adjusted OR 0.98, 0.97–0.99) (Fig. 1). The mortality rate in the ISS 50–75 group did not improve significantly (adjusted OR 0.96, 0.91–1.00) (Fig. 2). Age was a significant confounder for overall mortality (adjusted OR 2.44, 2.12–2.81) but was not significant for the ISS 50–75 group (adjusted OR 1.15, 0.64–2.07) (Table 2).

**Table 1:** Demographics (*hypothesis test comparing 16–49 to 50–75 group)

	Overall	16–49	50–75	*p*-value*
Number	8462	8224	238	
Age (year, IQR)	47 (26–69)	47 (26–69)	39 (23–56)	< 0.0001
Age ≥ 55 (*n*, %)	3,421, 40.4%	3,353, 40.8%	68, 28.5%	0.0002
Sex (*n*, %)				> 0.2
Male	6102, 72.1%	5925, 72.0%	177, 74.4%	
Female	2359, 27.9%	2298, 27.9%	61, 25.6%	
Penetrating (*n*, %)	261, 3.1%	261, 3.2%	7, 2.9%	> 0.2
Transfer in (*n*, %)	2640, 31.9%	2640, 32.1%	60, 25.2%	0.024
Mortality (*n*, %)	814, 11.1%	814, 9.9%	123, 51.7%	

## Discussion

We hypothesized that despite our proven improvement in trauma system performance, that ISS 50–75 polytrauma mortality would remain unchanged. Overall trauma mortality fell over the study period with no significant change in ISS 50–75 mortality. This supports our hypothesis that the fall in overall trauma mortality is predominantly driven by the improvement in outcomes of less severely injured patients. We demonstrated the critical trauma population is small enough to be comprehensively audit and reflect on (one patient per month, on average) (see Table 2).

**Table 2:** Multiple logistic regression

	Overall	ISS 50–75
Year	0.980 (0.969–0.992)	0.955 (0.916–1.001)
Age ≥ 55	2.44 (2.12–2.81)	1.15 (0.64–2.07)
Male	0.96 (0.83–1.12)	0.62 (0.34–1.14)
Penetrating	1.47 (1.01–2.13)	5.43 (0.63–46.99)
Transfer	0.63 (0.54–0.74)	0.57 (0.31–1.03)
Constant	0.11 (0.01–0.14)	2.53 (1.20–5.31)

The risk of death from injury continues to fall in developed trauma systems. Quality improvement methodology in trauma uses two disparate methodologies. Preventable mortality review examines death that were statistically predicted to survive [11]. Epidemiological reporting relies on the total event rate, in this case mortality, being a measure of system performance [12]. For the latter to allow benchmarking, standardized reporting must occur. The use of a universally collected scoring system (AIS and thus ISS) allows for extraction of large data sets from long time period without retrospectively collecting physiological parameters, such as in the Revised Trauma Score (RTS) [13] and Trauma Injury Severity score (TRISS) [14]. It also avoids the complication of relying on admission GCS and respiratory rate in a population commonly intubated, sedated, and ventilated in the pre-hospital phase [15].

Reporting of trauma system performance within the ISS 50–75 poly-trauma population is sporadic. At our state level, ISS 40–75 is reported [9]. ISS 40–75 is a much larger more heterogeneous group; there is a 20% reduction in mortality rate between ISS 40 and ISS 50 (Table 3) [8]. Our 20-year ISS 50–75 mortality of 53% lags behind retrospective data from Wurm et al. with a reported mortality rate of 36% (95% CI 26–46) over the period of 2000 to 2005 among 88 patient with an ISS of 50–75 [16]. This is higher performance during a much shorter study period compared to our data. Their group was slightly less severely injured with a mean ISS of 56.8 vs 59.0, and having most of their patients (75%) in the less severely injured, ISS 50–60 range. Candefjord et al. reported ISS 50–75 as a relevant group for comparison but did not report raw mortality [17]. We reconfirmed that age is a significant cofactor in overall ISS > 15 mortality; however, this is driven by less severely injured patients with the ISS 50–75 group suffering no significant influence from age. This could make ISS 50–75 mortality a robust and practical comparator of trauma centers rather that requiring the reporting of risk-adjusted mortality which requires granular data analysis.

**Table 3:** Differences between ISS 40–75 and 50–75

	ISS 40–75	ISS 50–75
Number	487	238
Age (year, IQR)	35 (21–56)	39 (23–56)
Age ≥ 55 (*n*, %)	133 27.3%	68, 28.5%
Sex		
Male (%)	360, 73.9%	177, 74.4%
Female (%)	127, 26.1%	61, 25.6%
Mortality (*n*, %)	206, 42.3%	123, 51.7%

This study was performed at a single center, albeit the largest volume in the state. Despite this, there was still considerable variability given the smaller number of cases. This may limit the usefulness of ISS 50–75 mortality as a reporting measure for trauma centers and limits the statistical power of the subgroup. There may be a true improvement in this group that has not been detected due to the high variability year-on-year, and small number of these severely injured patients over time. The injuries were almost exclusively blunt trauma given the low rates of stabbings and firearm-related injuries in Australia [18]. The performance of pre-hospital systems was incompletely measured with no ability to capture pre-hospital deaths in this dataset; this has been studied previously by our group. In this one year prospective study of all traumatic deaths, a majority of deaths occurred in the pre-hospital setting, most of these deaths were from central nervous system causes [19]. Centers with shorter pre-hospital times may experience more in-hospital deaths, biasing their ISS 50–75 results. This dataset is limited in its lack of physiological and early treatment data, and comprehensive adjustment for medical comorbidities as confounders for mortality. Outcome data are limited to only in-hospital mortality, rather than more nuanced measures of functional outcome [20].

The ‘critical polytrauma’ group represents the most severely injured patients a trauma system cares for. Despite representing only 3% of all major trauma patients, they represent 11% of all major trauma deaths. The sample is practical to capture with approximately 11 patients per year in a single trauma center—representing a group that could be extensively studied to identify opportunities for improvement in a mature trauma system. The reduction in mortality in the major trauma population is disproportionately driven by a reduction in less severely injured patients. Critical polytrauma mortality did not improve significantly, despite an overall improvement in mortality.
